# Diffusion-Weighted Imaging (DWI) derived from PET/MRI for lymph node assessment in patients with Head and Neck Squamous Cell Carcinoma (HNSCC)

**DOI:** 10.1186/s40644-020-00334-x

**Published:** 2020-08-08

**Authors:** Omar Freihat, Tamas Pinter, András Kedves, Dávid Sipos, Zsolt Cselik, Imre Repa, Árpád Kovács

**Affiliations:** 1 Doctoral School of Health Sciences, University of Pécs, P.O. Box: 7621, Vorosmarty 4, Pecs, Hungary; 2 Dr. József Baka Diagnostic, Radiation Oncology, Research and Teaching Center, Kaposvár, Hungary; 3 Medicopus Non-Profit Ltd., “Moritz Kaposi” Teaching Hospital, Kaposvár, Hungary; 4 Department of Medical Imaging, Faculty of Health Sciences, University of Pécs, Pécs, Hungary; 5 Oncoradiology, Csolnoky Ferenc County Hospital, Veszprém, Hungary; 6 Department of Oncoradiology, Faculty of Medicine, University of Debrecen, Debrecen, Hungary

**Keywords:** MRI, DWI, ADC, HNSCC, Metastasis, Benign, Lymph nodes

## Abstract

**Background:**

To determine the usefulness of Diffusion Weighted Imaging (DWI) derived from PET/MRI in discriminating normal from metastatic lymph nodes and the correlation between the metastatic lymph nodes with the grade and the localization of the primary tumor.

**Methods:**

Retrospective study of 90 lymph nodes from 90 subjects; 65 patients who had proven histopathological metastatic lymph nodes from (HNSCC) who had undergone ^18^F- PET/MRI for clinical staging and assessment and twenty-five lymph nodes were chosen from 25 healthy subjects. Apparent Diffusion Coefficient (ADC) map was generated from DWI with *b* values (0 and 800 s/mm^2^). ADC values of the metastatic lymph nodes were calculated and compared to the normal lymph nodes ADC values, ROC was used to determine the best cut-off values to differentiate between the two group. Metastatic lymph nodes ADC mean values were compared to primary tumor grade and localization.

**Results:**

ADCmean value of the metastatic lymph nodes in the overall sample (0.899 ± 0.98*10^− 3^ mm^2^/sec) was significantly lower than the normal lymph nodes’ ADCmean value (1.267 ± 0.88*10^− 3^ mm^2^/sec); (*P* = 0.001). The area under the curve (AUC) was 98.3%, sensitivity and specificity were 92.3 and 98.6%, respectively, when using a threshold value of (1.138 ± 0.75*10^− 3^ mm^2^/sec) to differentiate between both groups. Significant difference was found between metastatic lymph nodes (short-axis diameter < 10 mm), ADCmean (0.898 ± 0.72*10^− 3^ mm^2^/sec), and the benign lymph nodes ADCmean, (*P* = 0.001). No significant difference was found between ADCmean of the metastatic lymph nodes < 10 mm and the metastatic lymph nodes > 10 mm, ADCmean (0.899 ± 0.89*10^− 3^ mm^2^/sec), (*P* = 0.967). No significant differences were found between metastatic lymph nodes ADCmean values and different primary tumor grades or different primary tumor localization, (*P* > 0.05).

**Conclusion:**

DWI-ADC is an effective and efficient imaging technique in differentiating between normal and malignant lymph nodes, and might be helpful to discriminate sub-centimeters lymph nodes.

**Trial registration:**

The trial is registered in clinical trials under **ID:**NCT04360993, registration date: 17/04/2020.

## Introduction

Worldwide, head and neck cancer is the sixth most common malignancy, accounting for approximately 6% of all cancer cases and an estimated 1–2% of all cancer deaths [1]. H&N cancers are a heterogeneous group of cancers existing anatomically close to each other, but are different in terms of etiology, histology and diagnostic and treatment approaches [2]. About 91% of all H&N cancers are squamous cell carcinomas, 2% are sarcomas and the other 7% are adenocarcinomas, melanomas and not well-specified tumors [3].

Metastatic lymph nodes in head and neck tumors are malicious prediction factors tending to worsen the prognostics of patients with head and neck neoplasms, the accurate detection and diagnosis will help to optimize the treatment outcomes [4]. Due to the limitations of the invasive medical interventions (biopsy) which can’t always detect heterogeneity of intra-tumor structures from a single attempt, as well as non-rational to perform multiple biopsies for the patient or performing biopsies for all suspected lesions in the individual, [5] more realistic diagnostic methods were needed to provide a more inclusive view of tumor texture and biology including internal tumor characteristics. Previous studies have shown that multiparametric imaging can provide precise data for tumor biology and heterogeneity [6, 7]. CT and MRI are very useful imaging modalities in the initial assessment and diagnosis of head and neck cancers and it has been widely used for therapy delineation as well as surveillance and post-therapy follow up [8–10]. Morphological characteristics include size, shape, internal biological components and vascularity are important factors associated with the metastatic lymph nodes; although, the accuracy has some limitations [9, 11].. Hybrid Imaging (PET/CT & PET/MRI), on the other hand, was proposed to provide a more accurate and promising non-invasive alternative [5, 12–14].

MRI as single scan or combined with PET scan offers a verity of techniques for tissue assessment and intracellular characteristics in Oncology, this includes PET imaging parameters, MR Spectroscopy (MRS), Intravoxel Incoherent Motion (IVIM), ﻿Diffusion Tensor Imaging (DTI), ﻿Diffusion Kurtosis Imaging (DKI) as well as DWI represented by Apparent Diffusion Coefficient (ADC) [15, 16]. DWI as one of these offered techniques by PET/MRI is a non-invasive examination imaging technique allowing for the characterization of tissues based on the water molecule’s displacement motion (Brownian motion), [17–19] the range of motion is distinguished by its Apparent Diffusion Coefficient (ADC) values [20]. The signal loss in the diffusion sequences is caused by the water molecule’s water motion which causes phase dispersion of the spin, and the ADC map can measure the amount of signal loss within the biologic tissue [21, 22]. Clinically, there is a periodic and crucial question of whether malignant and normal lesions can be distinguished by DWI/ADC [23]. In general, it has been shown that benign lymph nodes have higher ADC values than malignant ones. Nevertheless, for daily use, there is a need for sensible threshold values to help physicians to determine whether the node is malignant or benign regardless of the invasive procedures followed to determine the nature of these nodes. DWI/ADC offers this non-invasive medical intervention. For example, a study by Das et al. was reported that ﻿1.791 × 10 − ^3^ mm^2^/s ADC cut off value in Sinonasal lesions was able to differentiate between benign and malignant lesions ﻿with 80% sensitivity and 83.3% specificity [24]. Barchetti et al. in a study of cervical lymph nodes in patients with HNSCC reported a cut off value of 0.965 × 10^− 3^ mm^2^/sec to differentiate between benign and malignant nodes with a sensitivity of 97%, a specificity of 93, 92% accuracy, 95% PPV, and 96% NPV of [25]. Wendl et al. in their study of Oral Squamous Cell Carcinoma (OSCC) reported an ADC value of 0.994 × 10^− 3^ mm^2^/sec as the best threshold with a sensitivity of 80%, a specificity of 65, 31% PPV, 93% NPV to discriminate between benign and malignant lymph nodes [26]. Suh et al. their meta-analysis suggested that the median ADC cutoff value of 0.965 × 10^− 3^ mm^2^/s can differentiate between benign and malignant nodes [27]. Most recent meta-analysis indicates that the studies which previously used DWI/ADC for differentiating benign from malignant nodes reported limited role due to small sample studies, a wide range of ADC threshold values [28]. The study suggested that ﻿it may be only lesions with mean ADC values above 1.75 × 10^− 3^ mm^2^/s are probably benign [28].

Our work has been built to distinguish between normal and malignant lymph nodes based on the ADC values and validate the standard ADC threshold value for clinical use. As well as to correlate the lymph nodes’ ADC values with the grade and the primary tumor localization.

## Material and methods

### Patients

A retrospective study was approved by the Clinical Center, Regional and Local Research Ethics Committee (CCRLREC), Doctoral School of Health Sciences, University of Pecs, and Somogy Megyei Kaposi Mor Educational Hospital, Pecs, Hungary. Approval number (IG/00686–000/2020). Requirement of the informed consent was waived and confirmed by the (CCRLREC) due to the retrospective nature, and all methods were carried out in accordance with the relevant guidelines and regulations (Declaration of Helsinki). From April 2016 to July 2019, 65 patients were recruited with confirmed primary HNSCCs underwent ^18^F-FDG PET/MRI (3 T) for staging and clinical assessment. All 65 patients were confirmed with metastatic lymph nodes due to HNSCC (time between imaging scan and biopsy ranged between 1 and 3 days), and 25 healthy subjects were randomly chosen from the radiology department available database, one node was selected from the neck region for evaluation. The inclusion criteria used for the patients in the study were: (1) Confirmed primary HNSCC malignancy by biopsy; (2) Multiparametric MR imaging (DWI); (3) PET/MRI for initial staging prior to primary treatment (Surgery or/and radio-chemotherapy); (4) Histopathological results were available for comparison; (5) No previous neck surgery, chemotherapy or (chemo)-radiotherpay. The criteria used for choosing healthy subjects were: (1) Free history of malignancy; (2) No previous neck surgery; (3) No previous treatment by chemo-radiotherapy; and (4) No head and neck lesions, inflammation or abscess or any abnormalities.

**Fig. 1:**
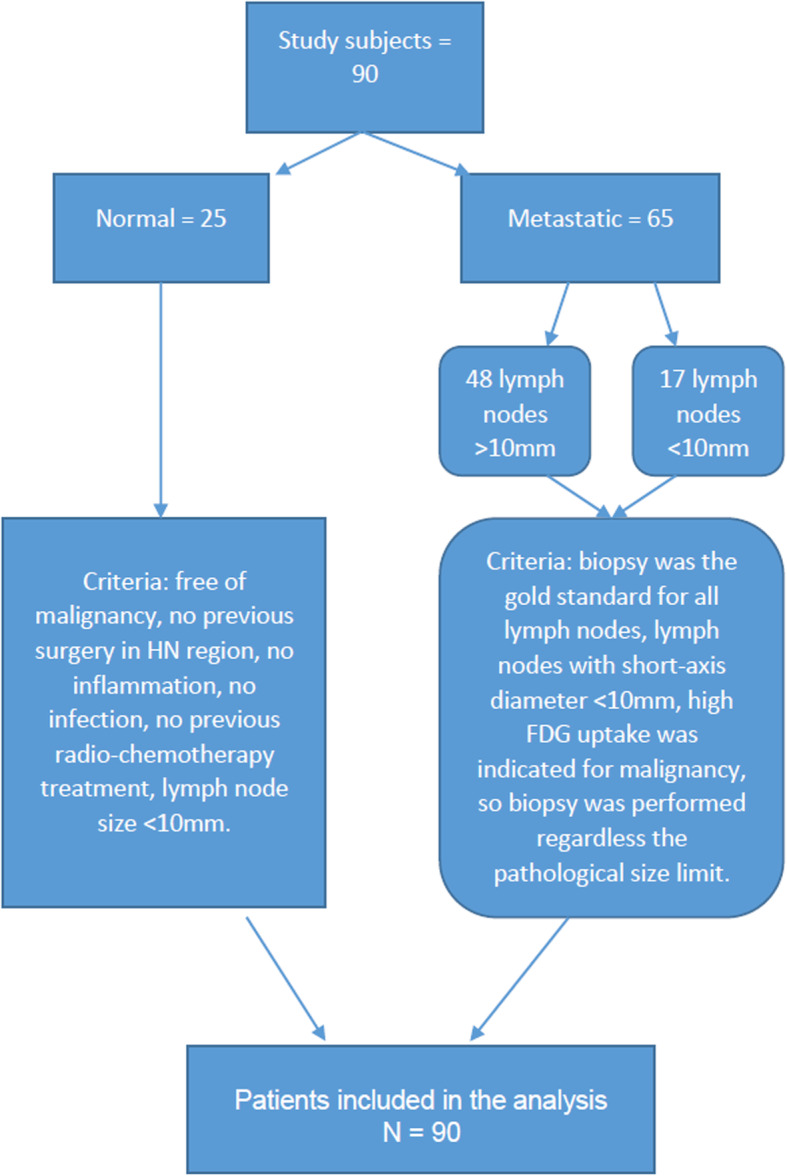
flowchart of the included subjects and the criteria used

The criteria used for considering lymph nodes as metastatic was confirmed after (1) Biopsy (gold standard); (2) High FDG accumulation was considered as indication for malignancy in the < 10 mm group (biopsy/FNA were taken). Metastatic lymph nodes with short-axis diameter < 10 mm (*n* = 17) and short-axis diameter > 10 mm (*n* = 48). The criteria used for considering lymph nodes as normal were: (1) Free history of malignancy; (2) Ovoid or smooth in shape; and (3) Short-axis diameter < 10 mm. Fig. 1.

Sample demographics were as follow: 65 patients with proven primary HNSCC and 25 healthy subjects. The histological grade of the primary tumor distribution over the primary tumor localization was as follow; well differentiated (G1) (*n* = 10) was in Oropharyngeal (n = 1), Hypopharyngeal (n = 1), Oral (*n* = 6), Sinus (*n* = 2); moderately differentiated (G2) (*n* = 35) was in Oropharyngeal (*n* = 7), Hypopharyngeal (*n* = 4), Oral (*n* = 11), and Laryngeal (n = 11); poorly differentiated (G3) (*n* = 25) was in Oropharyngeal (n = 4), Hypopharyngeal (*n* = 5), Oral (n = 7), and Laryngeal (n = 7). Patients demographics description in (Table 1).

**Table 1:** Subjects’ demographics

Characteristics	Value
Number of lymph nodes	**90**
Metastatic lymph nodes	65 (72.2%)
Benign lymph nodes	25 (27.7%)
Mean age (year)	
Baseline (healthy subjects)	53.4 ± 4.1 (37–64)
Metastatic disease patients	62.3 ± 8.3 (41–82)
Sex (overall)	
Male	59 (65.6%)
Female	31 (34.4%)
Primary HNSCC characteristics	
Baseline	25 (27.7%)
*Pharyngeal SCC*	22 (24.4%)
Oropharyngeal SCC	12 (13.3%)
Hypopharyngeal SCC	10 (11.1%)
*Laryngeal SCC*	18 (20.0%)
*Oral carcinoma*	23 (25.6%)
*Sinus carcinoma*	2 (2.2%)
*Pharyngeal SCC*	27 (29.3%)
Tumor grade	
Baseline	25 (27.7%)
Well differentiated	9 (10.0%)
Moderately differentiated	34 (37.8%)
Poorly differentiated	22 (24.4%)
T category	
Baseline	25 (27.7%)
T1	8 (8.7%)
T2	27 (30.0%)
T3	30 (33.3%)
T4	25 (27.8%)
N category	
Baseline	25 (27.7%)
N1	14 (15.6%)
N2	43 (46.7%)
N3	8 (8.9%)
M category	
Baseline	25 (27.7%)
M0	56 (62.3%)
M1	9 (10.0%)

### Imaging protocols

An integrated PET/MRI (3 T magnetic field strength) was performed for the head and neck region by OEM neck coil with 16 channels of the head/neck for both conventional and diffusion-weighted MR to cover lymph nodes extending from the skull base to the thoracic outlet. The sequence included a 3D volumetric interpolated breath-hold T1 weighted sequence on the transverse plane and T2 turbo spin-echo imaging (T2WI TSE) on the transverse plane, axial Dixon FS T1-weighted TSE sequence and a coronal TSE Dixon FS sequence on the coronal plane and T1 weighted FS on the axial and coronal plane.

DWI sequence was performed on the axial plane with *b* values of 0 and 800mm^2^/s [9, 25, 29]. We have chosen two b values because different multiple b values didn’t affect the diagnostic performance [30, 31]. and 800mm^2^/s was the best choice to prevent loss of signal and avoiding image distortion which is usually observed at higher b values [32, 33], most of the previous studies used a range between 0 and 1000 mm^2^/s [34]. DWI pulse sequences were defined as follows: FoV 315 mm, repetition time (TR) 9900 ms, 5 mm slice thickness, voxel size 2.3 × 2.3 × 5 mm and slice gap 10 mm. The attenuation correction technique was used based on the Dixon sequence. Images were corrected and reconstructed with an iterative algorithm (21 subsets, 3 iterations, and a Guassian filter (3D iterative (OP-OSEM)) with a full width at half maximum (4 mm) for scatter correction, 172 × 172 matrix).

### Image analysis and data interpretation

The PET/MRI examinations were transferred to the main workstation and the patients were assessed by the physicians of the Oncoradiology team at the Baka Jozsef Oncoradiology center. The ADC map generated from the DWI was used for the measurement of the ADC values. In each patient with multiple metastatic lymph nodes, the largest lymph node was selected for evaluation. The nodal ADC values were obtained by drawing a region of interest (ROI) covering as much as possible of the most solid and/or homogenous part (most restriction in DWI image), [35] avoiding necrotic parts if the node was partially necrotic while full necrotic nodes were excluded from the study, and avoiding the outer contour of the node. The measurements were made on a single slice only [26, 29]. This method was used due its feasibility in the daily use, less time consuming and the results did not differ significantly when compared to other approaches (whole tumor approach for example) [36, 37].. “Avg” was the ADC mean average and “Dev” was the standard deviation which represented the homogeneity of the node tissue.

## Statistical analysis

SPSS version 25.0 (IBM Corporation, USA) was used for the statistical analysis. Continuous variables were compared using the Student’s t-test or analysis of variance and were expressed as the mean ± standard deviation for the variables with a normal distribution. The Independent sample t-test was used to compare the ADC mean values of the metastatic and the normal lymph nodes as well as between sub-groups analysis. ROC curve was applied to determine the sensitivity and specificity, AUC, and the optimal threshold to differentiate between normal and malignant nodes. The selection of the optimal threshold was chosen at the point with the highest Youden index (maximizing both sensitivity and specificity). The one-way analysis of variance (ANOVA) was applied to evaluate the coincidence between primary cancer’s grade with the metastatic lymph nodes’ ADC values and with the primary tumor localization. Post Hoc analysis (Scheffe) was used to compare the parameters in terms of histopathology in case of significant results.

## Results

**Fig. 2:**
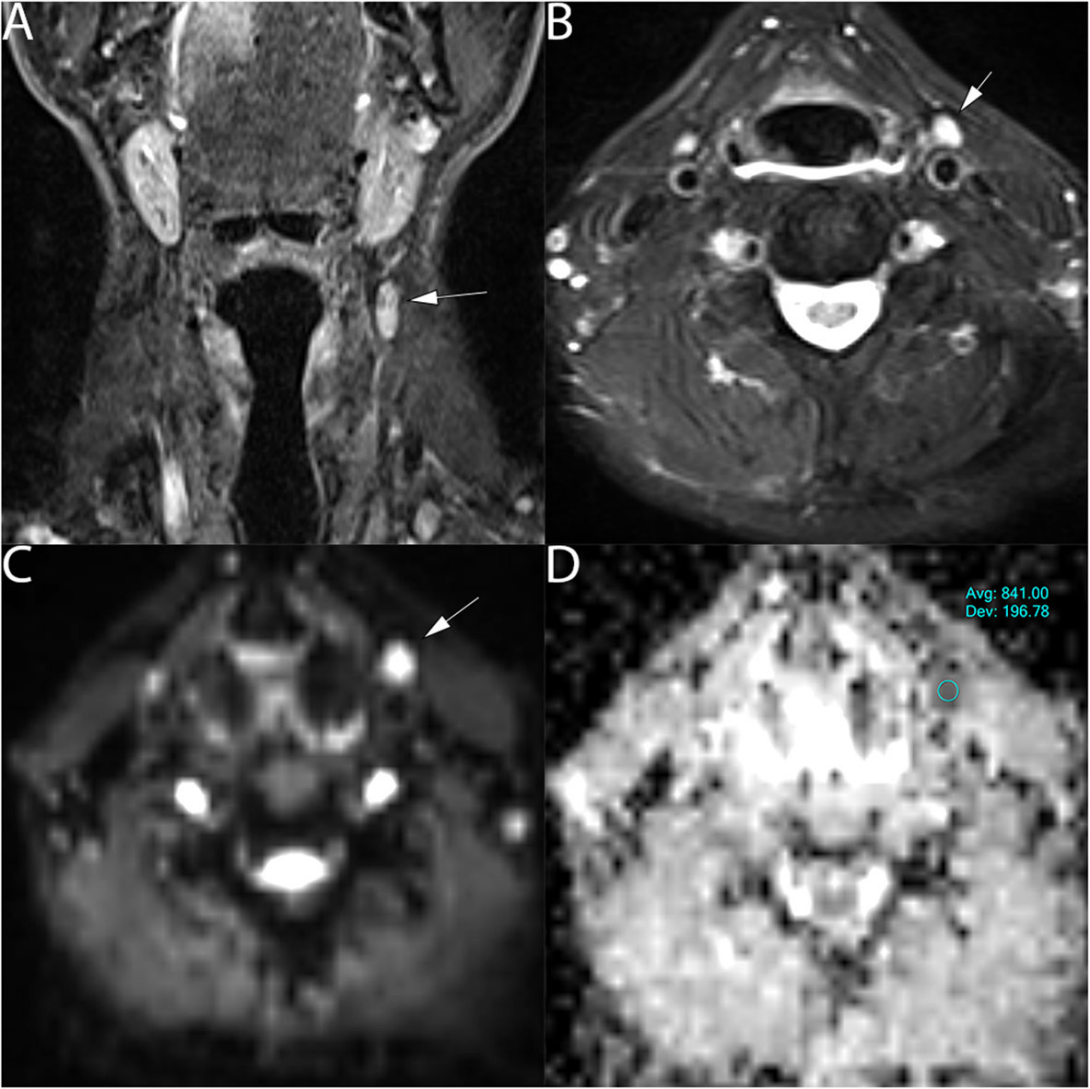
65 years old male with para-pharyngeal sub-centimeters lymph node positive malignancy from laryngeal SCC on the left side of the neck. **a** T2 coronal show the lymph node, (arrow). (B) T2 axial show the axial extension of the lymph node (6x8mm), (arrow) (**c**) DWI show hyperintense area at b value 800mm^2^/s (arrow) and (**d**) ADC map on the targeted lymph node show hypointense signal, an ADC value of (0.841 ± 0.19*10^− 3^ mm^2^/s)

**Fig. 3:**
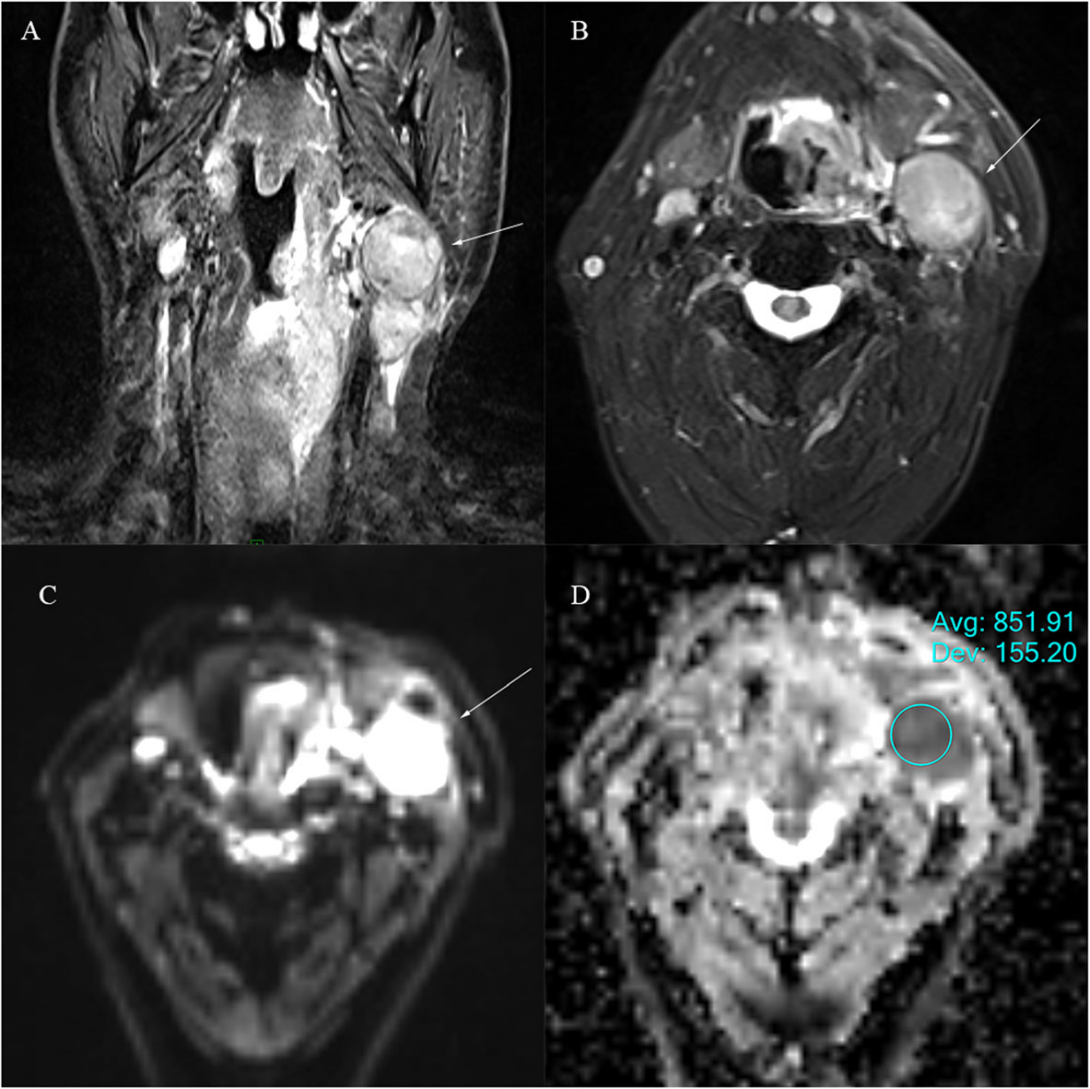
71 years old male with left positive para-pharyngeal enlarged lymph node from oropharyngeal SCC on the left side of the neck. **a** T2 coronal show enlarged lymph node axial dimensions, (34x35mm) (arrow). **b** T2 axial show the axial extension of the lymph node. **c** DWI show high signal at b value 800mm^2^/s and (**d**) the ADC map on the targeted lymph node show an ADC value of (0.851 ± 0.16*10^− 3^ mm^2^/s)

**Fig. 4:**
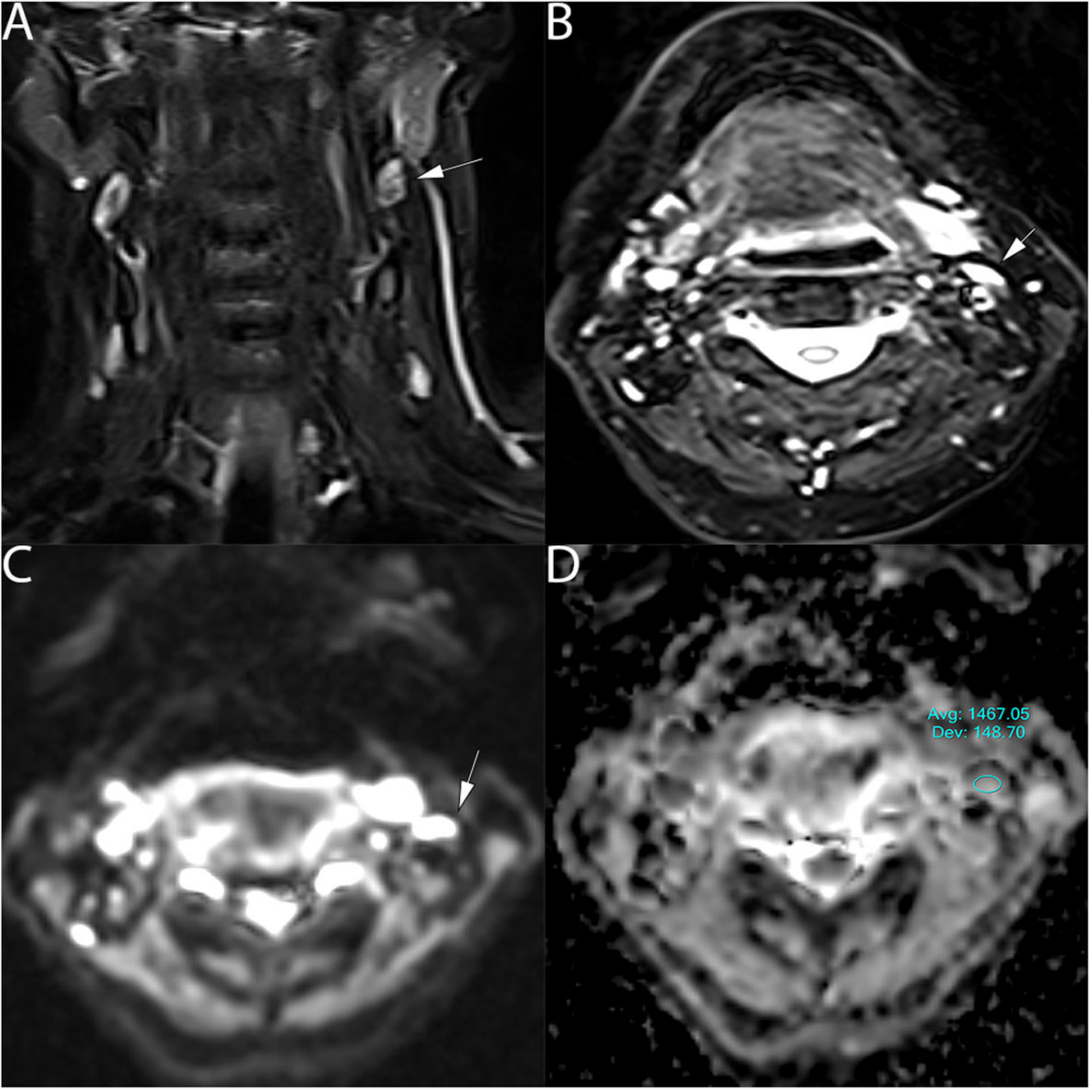
61 years old healthy male with normal lymph node in the left side of the neck. **a** T2 coronal show normal lymph nodes (arrow). **b** T2 axial view shows the axial extension of the left lymph node, (5x7mm), (arrow). **c** DWI show high signal at b value 800mm^2^/s (arrow) and (**d**) the ADC map on the targeted lymph node show an ADC value of (1.467 ± 0.15*10^− 3^ mm^2^/s) (arrow)

Overall, 90 lymph nodes were studied, 65 metastatic lymph nodes: minimum short-axis diameter size (6-44 mm) and maximum short-axis diameter size (7-85 mm), Figs. 2 and 3, were assessed and compared to 25 normal lymph nodes, minimum short-axis diameter size range (4-9 mm), and maximum short-axis diameter size range (6–13 mm), Fig. 4.

### The ADC values of normal and metastatic lymph nodes

**Fig. 5:**
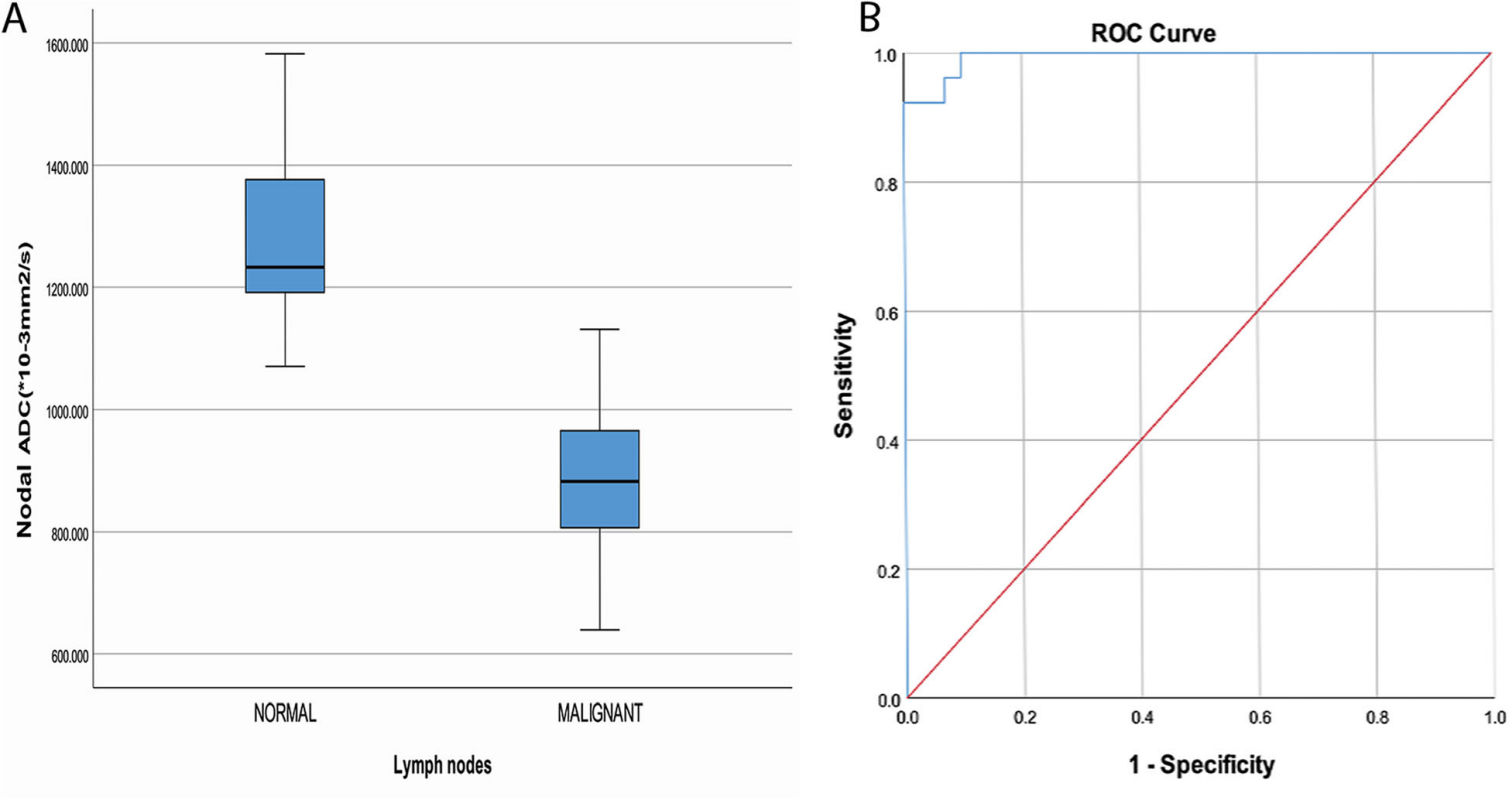
**a** Box-and-whisker plots of the normal and metastatic lymph nodes ADC values; significant difference was reported. *P* < 0.05. **b** ROC curve for the ADC value for differentiating between metastatic and normal

Biopsy was the gold standard for all metastatic lymph nodes confirmation, sub-centimeters lymph nodes < 10 mm were first assessed by PET/MRI, lymph nodes that show high FDG uptake were considered for biopsy regardless the pathological size limit, malignancy confirmed lymph nodes by biopsy then considered in the study. According to the statistical analysis after comparing ADC values of the metastatic lymph nodes with ADC values of the normal lymph nodes, the ADCmean value of metastatic lymph nodes (0.899 ± 0.98) was significantly lower than the ADCmean value of normal lymph nodes (1.267 ± 0.88), (*P* = 0.001), Fig. 5 A.

To obtain the optimal ADC value to differentiate between metastatic and normal nodes, we applied the ROC curve. The result shows that when using (1.138 ± 0.75) as an optimal threshold value to differentiate between metastatic and normal nodes, the AUC was 98.3%, sensitivity and specificity were 92.3 and 98.6%, respectively. Fig. 5 B.

**Fig. 6:**
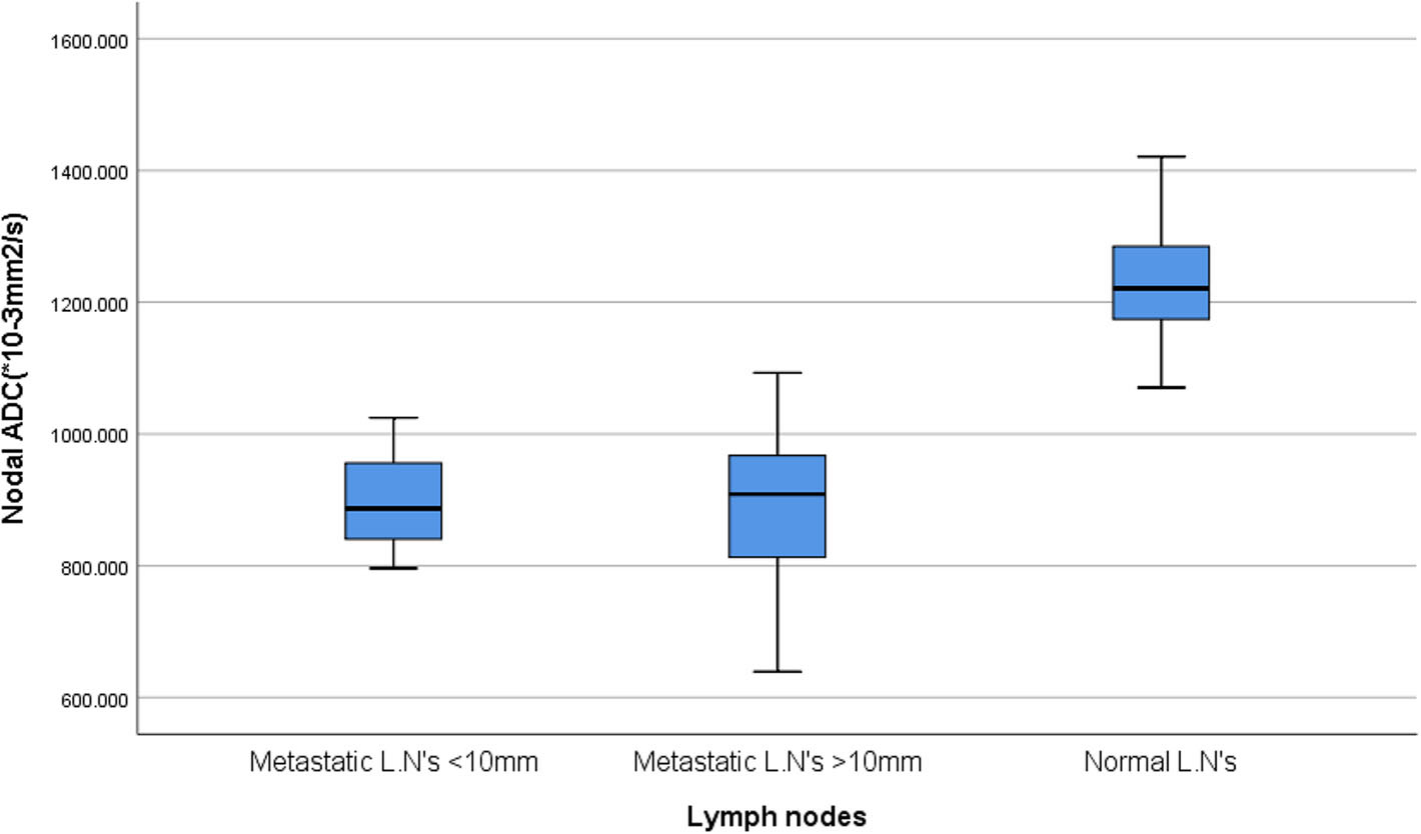
Box-and-whisker plots of the mean ADC values of the metastatic lymph nodes sub-grouped into (short-axis diameter < 10 mm and short-axis diameter > 10 mm) were significantly lower than normal lymph nodes ADC values (*P* = 0.001), no significant difference between metastatic lymph nodes with short-axis diameter < 10 mm and metastatic lymph nodes with short-axis diameter > 10 mm, (*P* = 0.967)

Furthermore, to assess the ability of DWI to discriminate small metastatic lymph, we divided the patients with metastatic lymph nodes into short-axis diameter < 10 mm (*n* = 17), and lymph nodes with short-axis diameter > 10 mm (*n* = 48) and compared to the ADCmean values with those for the control group (*n* = 25). The results show that ADCmean values of the metastatic lymph nodes short-axis diameter < 10 mm (0.898 ± 0.72) was significantly lower than ADCmean values of the normal lymph nodes, (*P* = 0.001). No significant difference found between ADCmean of the metastatic lymph nodes with size < 10 mm and the metastatic lymph nodes with size > 10 mm (0.899 ± 0.89), (*P* = 0.967). Fig. 6.

### Correlation between the primary HNSCC grade and metastatic lymph nodes’ ADC values

**Fig. 7:**
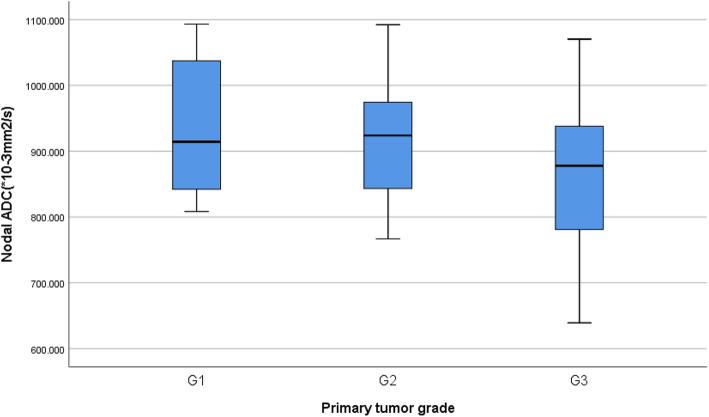
**a** Box-and-whisker plots of the lymph nodes ADC according to the primary tumor grades (G1-G3). No significant difference was found between the three grades, *P* > 0.05

Our results show that metastatic lymph nodes’ ADCmean values from poorly differentiated primary HNSCC was (0.863 ± 0.59) and the ADCmean values were (0.916 ± 0.84, 0.936 ± 0.11) for the metastatic lymph nodes from well and moderately differentiated, respectively. Although there was a trend toward decreasing ADC with increasing degree of differentiation, but significant result was not found, (*P* = 0.076). Table 2 and Fig. 7

**Table 2:** measured ADCmean and the statistical correlations

Parameter	ADCmean (*10^− 3^ mm^2^/s)	*P*-value
**Llymph nodes**		0.001*
Normal L.N’s	1.267 ± 0.88	
Metastatic L.N’s	0.899 ± 0.99	
**Primary tumor grade**		0.076
Well differentiated	0.936 ± 0.11	
Moderately Well differentiated	0.916 ± 0.84	
poorly Well differentiated	0.863 ± 0.59	
**Primary tumor localization**		0.431
Oropharyngeal	0.866 ± 0.93	
Hypopharyngeal	0.936 ± 0.18	
Laryngeal	0.898 ± 0.15	
Sinus carcinoma	0.879 ± 0.54	
Oral cavity	0.903 ± 0.95	

*. Significant at level of *P* < 0.05

.

### The ADC values of lymph nodes and the correlation with the primary HNSCC tumor localization

**Fig. 8:**
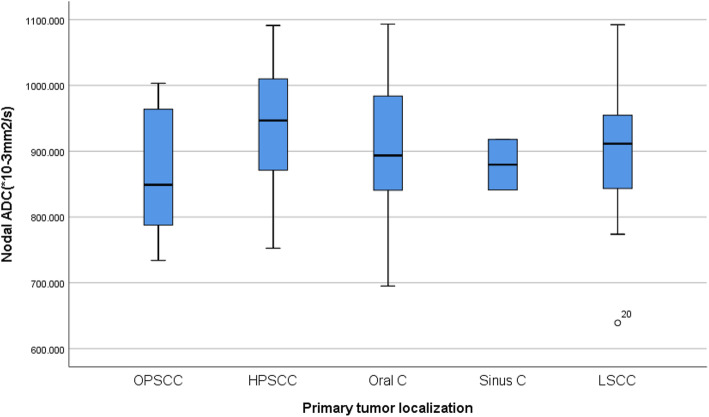
Box-and-whisker plots of the lymph nodes ADC according to the primary tumor localization. No significant differences were reported, *P* > 0.05

ADCmean values of the metastatic lymph nodes from OPSCC, HPSCC, LSCC, Sinus carcinoma and Oral cavity carcinoma were calculated and assessed, Table 2. ANOVA test was used to compare the groups’ mean value differences; ADCmean values were (0.866 ± 0.93, 0.936 ± 0.18, 0.898 ± 0.15, 0.879 ± 0.54, and 0.903 ± 0.95), respectively. No significant difference was found (*P* = 0.431). Table 2 and Fig. 8.

## Discussion

In our study, we found a statistically significant difference between metastatic and normal lymph nodes’ ADC values (*P* = 0.001); the result was in agreement with previous authors [19, 38–41]. Moreover, our study proposes that the best threshold to differentiate between normal and malignant nodes was (1.138 + 0.75*10^− 3^ mm^2^/sec), with (92.3%) sensitivity and (98.6%) specificity. Our study shows that DWI might be helpful to discriminate sub-centimeters metastatic lymph nodes.

The characteristics of the malignant tumors in comparison to the benign lesions were the tendency of the malignant tumors to have high cellularity, large nucleus to cytoplasmic ratio, less intercellular space, and a larger number of intercellular organelles than the benign lesions [42]. In general, malignant cancers are hyperchromatic, associated with nuclei, and show hypercellularity [43]. These features explain why the water molecules are more likely to be restricted in malignant tumors in comparison to benign lesions [42]. Moreover, accurate differentiation can prevent patients with non-cancerous lymph nodes from unnecessary procedures [44]. In addition, the differentiation between the metastatic and benign lymph nodes remain challenging when taking into account that none of the morphological characteristics, such as lymph node size, shape or availability of necrosis is definitely reliable [45].

Our result was similar to other authors, for example; Vandecaveye et al. reported that the best threshold to differentiate between benign and malignant lymph nodes was 0.94*10^−^ 3 mm^2^/sec [46]. de Bondt et al. found that the best threshold to differentiate between benign and malignant lymph nodes was 1.0*10^−^ 3 mm^2^/sec [47]. Perrone et al. were found that a cut-off value of 1.03*10^− 3^ mm^2^/s was the best to differentiate between benign and malignant tumors in their study of the cervical lymph nodes in HNSCC [38].

It’s also questionable whether DWI can provide valuable information when measuring sub-centimeters lymph nodes (< 10 mm), in our study we found that DWI might be useful for discriminating small lymph. According to Barchetti et al. DWI was efficient to differentiate small metastatic lymph nodes in HNSCC, (94.6%) of the measured lymph nodes were < 10 mm in size, a threshold of 0.96510^− 3^ mm^2^/sec was used as best cut-off value [25]. De Bondt et al. found a similar result, DWI was able to differentiate metastatic from benign lesion in HNSCC (95.4% of the measured lymph nodes were < 10 mm) using an optimal ADC threshold of 1.0 × 10^− 3^ mm^2^/s [47]. Although, other authors found that DWI does not allow to differentiate small metastatic lymph nodes in HNSCC [48].

It has been suggested that the cellularity of the metastatic lymph nodes might be similar to those of the primary tumor, therefore, it was proposed that the ADC values from metastatic lymph nodes from highly and moderately differentiated carcinomas may exhibit higher ADC values than those from poorly differentiated carcinomas [45]. Thus, poorly differentiated (G3) malignancies which are characterized by high mitotic activity will result in a high nucleus cytoplasmic ratio, be comparatively smaller in size, and have higher cellularity of the cells compared to well-moderately differentiated ones (G1, G2) [49]. Therefore, high-grade malignancies tend to have more restricted diffusion than low-grade malignancies [49]. These clinical suggestions have been studied and proven after having been tested and documented as reliable findings [50].

The results from our study of comparing the metastatic lymph nodes’ ADC values with the primary tumor grade showed that the ADC values of the lymph nodes from different primary HNSCC tumor grades were not significantly different when compared to the lymph nodes from the G1 and G2 primary HNSCCs. This was in agreement with *King* et al. when reported that there was no significant correlation between the ADC values of the metastatic lymph nodes with the grade of the primary tumor [51]. *Nakamatsu* et al. also found no statistical differences in the ADC values of enlarged lymph nodes from the three histological grades of the primary tumors [52]. On the other hand, some studies reported that metastatic lymph nodes ADC values were significantly different when compared to the primary tumor grades [45, 53]. Which means that metastatic lymph nodes from higher tumor grade (G3) shows lower ADC values [45, 53]. However, our result was controversial to these reports.

We have assessed the metastatic lymph nodes to determine the accuracy of DWI in discriminating lymph nodes from different primary tumor localization. Although the ADC values showed variations, no significant differences were found.

The study’s limitations include; First, the heterogeneity of the sample, which means multiple primary tumor localization. Second, the result may not be valid for all health centers due to the variation of MRI technologies and b-value strengths (the accuracy and quality of the magnetic field depend on the vendor). Third, only one slice measurements were applied, which may represent weakness in the study, although, we applied this method because it easier to perform, less time consuming and does not differ significantly from other approaches [36, 37]. Fourth, retrospective design of the study.

## Conclusion

Our study confirms the feasibility of DWI in differentiating between normal and metastatic lymph nodes. It’s also might be useful to differentiate sub-centimeters lymph nodes.

## Data Availability

The datasets generated during and/or analysed during the current study are available in the [clinical trials] repository, [https://register.clinicaltrials.gov/prs/app/action/ViewOrUnrelease?uid=U00051EQ&ts=6&sid=S0009QZD&cx=qiwsbh

## Abbreviations

DWI: Diffusion Weighted Imaging
ADC: Apparent Diffusion Coefficient
AUC: Area Under the Curve
ROI: Region of Interest
PET/MRI: Positron Emission Tomography/Magnetic Resonance Imaging
PET/CT: Positron Emission Tomography/Computed Tomography
HNSCC: Head and Neck Squamous Cell Carcinoma
HPSCC: Hypopharangeal Squamous Cell Carcinoma
LSCC: Laryngeal Squamous Cell Carcinoma
NPC: Nasopharyngeal Carcinoma
OPSCC: Oropharyngeal Squamous Cell Carcinoma
AJCC: American Joint Committee on Cancer
